# High-content siRNA 3D co-cultures to identify myoepithelial cell-derived breast cancer suppressor proteins

**DOI:** 10.1038/s41597-021-00924-9

**Published:** 2021-05-28

**Authors:** Hendrika M. Duivenvoorden, Natasha K. Brockwell, Cameron J. Nowell, Kaylene J. Simpson, Belinda S. Parker

**Affiliations:** 1 Department of Biochemistry and Genetics, La Trobe Institute for Molecular Science, La Trobe University, Melbourne, 3086 Australia; 2 School of Biological Sciences, Monash University, VIC Clayton, 3168 Australia; 3 Cancer Immunology Program, Peter MacCallum Cancer Centre, Melbourne, VIC 3000 Australia; 4 Sir Peter MacCallum Department of Oncology, University of Melbourne, Parkville, VIC 3052 Australia; 5 Drug Discovery Biology, Monash Institute of Pharmaceutical Sciences, Monash University, Melbourne, VIC 3052 Australia; 6 Victorian Centre for Functional Genomic, Peter MacCallum Cancer Centre, Melbourne, VIC 3000 Australia

**Keywords:** Breast cancer, Prognostic markers, Mechanisms of disease

## Abstract

Understanding how cancer cells interact with the surrounding microenvironment early in breast cancer development can provide insight into the initiation and progression of invasive breast cancers. The myoepithelial cell layer surrounding breast ducts acts as a physical barrier in early breast cancer, preventing cancer cells from invading the surrounding stroma. Changes to the expression profile and properties of myoepithelial cells have been implicated in progression to invasive carcinoma. Identifying the molecular drivers of myoepithelial cell-mediated tumour suppression may offer new approaches to predict and block the earliest stages of cancer invasion. We employed a high-content approach to knock down 87 different genes using siRNA in an immortalised myoepithelial cell line, prior to co-culture with invasive breast cancer cells in 3D. Combined with high-content imaging and a customised analysis pipeline, this system was used to identify myoepithelial proteins that are necessary to control cancer cell invasion. This dataset has identified prospective myoepithelial suppressors of early breast cancer invasion which may be used by researchers to investigate their clinical validity and utility.

## Abstract (summary)

**Table:** 

Measurement(s)	cell proliferation assay • Myoepithelial Cell • cell viability assay • protein expression assay
Technology Type(s)	cell counting technique • high content screen of cells treated with library of siRNAs • imaging technique • western blot analysis
Factor Type(s)	gene targeted by siRNA
Sample Characteristic - Environment	cell line

Machine-accessible metadata file describing the reported data: 10.6084/m9.figshare.14533644

## Background & Summary

Breast cancer that is confined to the ductal system (ductal carcinoma *in situ*, DCIS) has a 5-year survival rate of 99%. However, it is estimated that 14–53% of untreated DCIS will progress to invasive disease, supporting additional treatment to reduce the impact of disease and the risk of subsequent spread to the lymph nodes and distant organs^1,2^. The myoepithelial cells and basement membrane constitute the ‘boundary’ of the breast ducts and it is the presence of myoepithelial cells that is a distinguishing factor between DCIS and invasive ductal carcinoma (IDC). Whilst tumour cells in DCIS and IDC appear transcriptionally similar, expression profiling of myoepithelial cells derived from DCIS or normal breast tissue have revealed distinct changes that suggest myoepithelial cells may play an important role in dictating the DCIS to IDC transition^3,4^. However, few models exist with which to examine the interaction between myoepithelial and breast cancer cells to dissect the functional implications of such changes.

The laboratory has developed a 3D cell culture platform, whereby interactions of tumor and myoepithelial cells can be monitored within a reconstituted basement membrane. Using this 3D model, we have previously revealed that myoepithelial cell interactions are sufficient to suppress tumour cell invasion, reverting invasive tumour cell growth to a less invasive, DCIS-like state^5^. Furthermore, our investigations using this 3D co-culture system have revealed myoepithelial proteins that are essential for blocking tumor invasion, the suppression of which allows tumor invasion despite the physical presence of myoepithelial cells^5^. One such protein is the cysteine protease inhibitor stefin A (also known as cystatin A, CSTA), with myoepithelial-specific knockdown of stefin A allowing breast cancer cell invasion in 3D culture^5^. Based on results seen in 3D culture we proposed that stefin A could be a candidate biomarker to stratify patients into high risk and low risk invasive recurrence groups. Interrogation of DCIS clinical cohorts revealed that stefin A expression was reduced and lost in myoepithelial cells surrounding high grade DCIS, patients most at risk of progression to IDC. These data validated use of the 3D co-culture model to study the interactions between tumor and myoepithelial cells to identify clinically relevant regulators of breast cancer progression and potential biomarkers to predict risk of invasive recurrence.

One proposed mechanism of progression of DCIS to IDC is the loss of surrounding myoepithelial cells. There is little known about how or why myoepithelial cells disappear in IDC, however it has been suggested that a loss of certain proteins, reduced cell to cell contact, or degradative enzymes cause myoepithelial cell death^6^. Cell-cell adhesion is important for the normal functioning of cells, tissues and organs, and the loss of cell adhesion is an early step in tumour development^7^. The unique organization of the breast architecture whereby myoepithelial cells surround luminal epithelial cells^8^ allows for a unique opportunity to examine the role of myoepithelial adhesion proteins in tumorigenesis.

**Fig. 1:**
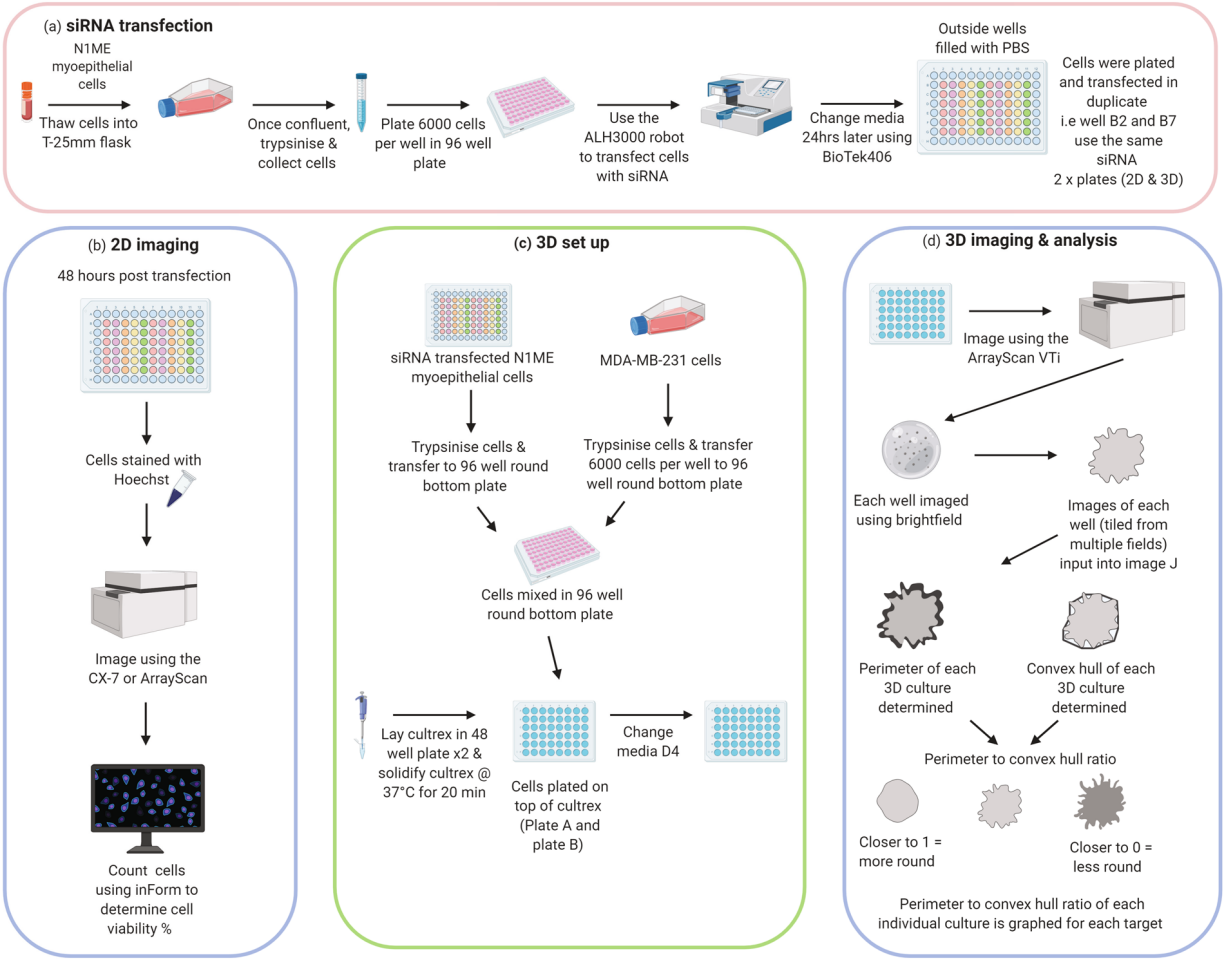
Workflow for high content 3D co-culture. SMARTpool siRNA transfection (**a**) Cells were cultured to ~70–80% confluence, trypsinised and plated at 6000 cells/well in a 96 well plate. Cells were reverse transfected in duplicate using the ALH300 robot at 40 nM final concentration of targets and controls using DharmaFECT 3. Using a BioTek406 liquid handling workstation, media was changed after 24 hours. (**b**) 48 hours post transfection, 1 plate of cells was stained with Hoechst and imaged using the CX7 or ArrayScan. Images were then run through inForm software to count nuclei to give an overall cell number per well. (**c**) At 48 hours post transfection the duplicate plate was trypsinised and contents of well transferred to a 96 well round bottom plate with 6000 MDA-MB-231 cells per well. Cultrex was laid in 48 well plates followed by seeding of N1ME & MDA-MB-231 cells from the 96 well round bottom plate. Media was changed on D4. (**d**) 3D cultures were stained with Hoechst and imaged using the ArrayScan. Tiled images were then run through an Image J script to determine the perimeter of each individual colony and the convex hull. This information generated the perimeter to convex hull ratio for each individual colony which is graphed to give the overall average ‘roundness’ of the 3D co-culture. Created with http://BioRender.com.

**Fig. 2:**
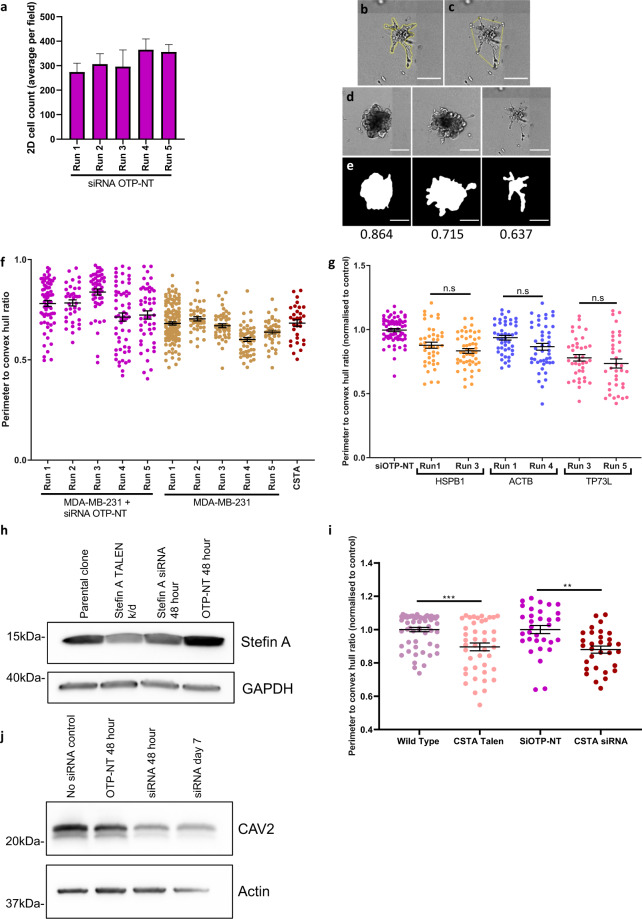
Validation of siRNA approach. (**a**) 2D cell count of Hoechst stained N1ME cells 48 hours post transfection with siOTP-NT across the 5 different screen runs (plates) conducted on different dates. Cell count was performed per field using inForm software. (**b**) Perimeter of an individual colony. (**c**) Convex hull of a colony. (**d**) Examples of colonies from least to most invasive. (**e**) Examples of mask generation for individual colonies and their corresponding perimeter to convex hull ratios. A value of 1 indicates a smooth object; as the value moves away from 1 towards 0, the number and/or size of the protrusions from the colony is increased. Scale bars represent 200 µm. (**f**) Perimeter to convex hull ratio of MDA-MB-231 cells in co-culture with N1ME cells following transfection with siOTP-NT or stefin A (CSTA) or MDA-MB-231 cells alone. Differences in the invasive growth of 3D cultures were determined by calculating the ratio between the perimeter and convex hull of each colony (circularity). Graph shown is raw data and represents variability in 3D cultures over 5 different plates conducted on different dates. (**g**) Perimeter to convex hull ratio of MDA-MB-231 cells in co-culture with N1ME cells following transfection with indicated siRNA. Data has been normalised to the siOPT-NT on the same run to indicate that comparison with in-plate controls results in non-significant differences between siRNA results across different plates/runs. Dots in the graph represent an individual 3D structure/colony growing within the well. Error bars represent SEM. (**h**) Western blot of stefin A and GAPDH expression in N1ME cells: parental clone, TALEN stefin A k/d (permanent knockdown), stefin A siRNA and siRNA OTP-NT at 48 hours post transfection. (**i**) Comparison of 3D circularity of MDA-MB-231 cells co-cultured with N1ME cells with stefin A knockdown via TALEN or siRNA reveals comparable stefin A knockdown phenotypes. (**j**) Western blot of CAV2 and actin expression in cells with no siRNA transfection, OTP-NT 48 hours post transfection, siRNA for CAV2 48 hours and 7 days post transfection.

**Fig. 3:**
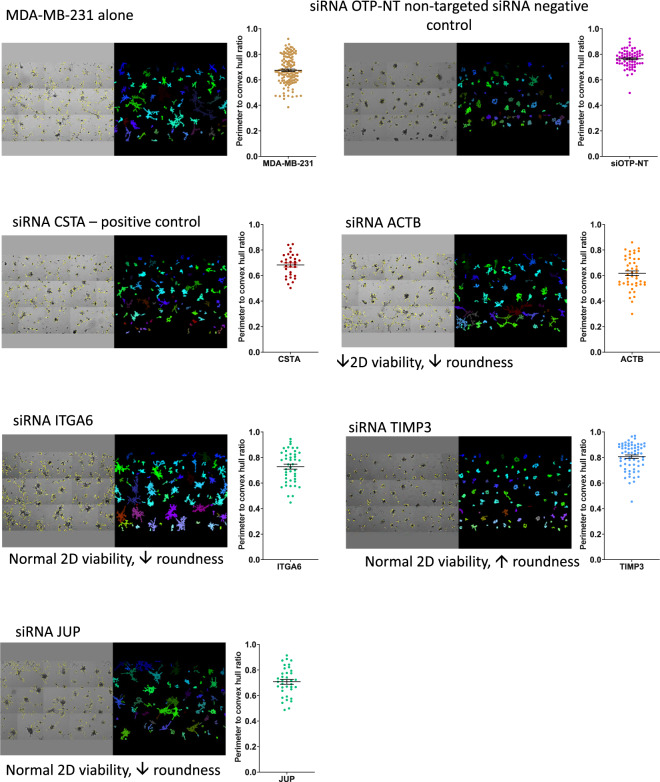
3D culture images and quantification using Fiji. 3D co-culture images taken using the ArrayScan microscope using a 5x objective were stitched together using HCS studio 3 software to give an overview of the entire well. Images were then run through Fiji to generate masks around individual cultures which were used to calculate the perimeter to convex hull ratio. Co-cultures shown are from single cell control, siRNA control, stefin A (CSTA) and various siRNAs which represent a range of observed phenotypic effects as indicated. Perimeter to convex hull ratio shown for the siRNA targets pictured. Data is raw and has not been normalised to the siOTP-NT in the run. Dots in the graph represent an individual 3D structure/colony growing within the well. Error bars represent SEM. siOTP is shown in purple, MDA-MB-231 cells alone are gold, siRNA which has resulted in no change in 2D cell viability but has reduced 2D ‘roundness’ is shown in green, siRNA has resulted in decreased 2D viability and 3D ‘roundness’ is shown in orange and siRNA which has resulted in no change in 2D cell viability but has increased 3D ‘roundness’ is shown in blue.

**Fig. 4:**
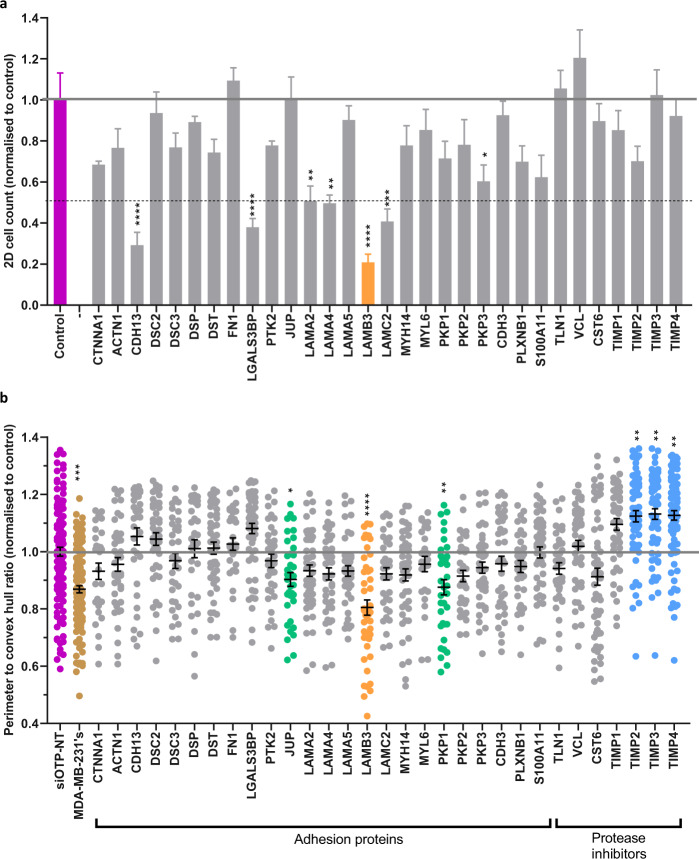
siRNA knockdown of adhesion proteins and protease inhibitors affects myoepithelial cell function. (**a**) Cell viability of N1ME cells cultured in 2D at 48 hours post-siRNA transfection normalised to the siOTP-NT control. Cell count was quantified per field using inForm software. Cell viability of <50% relative to the control is considered a significant impairment. Error bars represent SEM across technical replicates. (**b**) Perimeter to convex hull ratio of MDA-MB-231 cells grown in co-culture with N1ME cells following siRNA transfection. Differences in the invasive growth of 3D cultures were determined by calculating the ratio between the perimeter and convex hull of each colony (circularity). A value of 1 indicates a smooth object; as the value moves away from 1 towards 0, the number and/or size of the protrusions from the colony is increased. All data was normalised to the control, MDA-MB-231 cells co-cultured with N1ME cells transfected with siOTP-NT that was run on the same plate. Dots in the graph represent an individual 3D structure/colony growing within the well. Cultures that had a decrease in roundness from control but no alterations to 2D cell viability are represented in green. The culture that had a reduction in roundness from siOTP-NT control in 3D and induced an >80% reduction in 2D viability is shown in orange. siOTP is shown in purple, MDA-MB-231 cells alone are gold, siRNA which has resulted in no change in 2D cell viability but has reduced 2D ‘roundness’ is shown in green, siRNA has resulted in decreased 2D viability and 3D ‘roundness’ is shown in orange and cultures that resulted in an increase in roundness compared to control, without impacting 2D viability, are shown in blue. Statistical significance calculated compared to control (purple). *p < 0.05, **p < 0.01, ***p < 0.001, ****p < 0.0001. *n* = *2*. Error bars represent SEM.

**Fig. 5:**
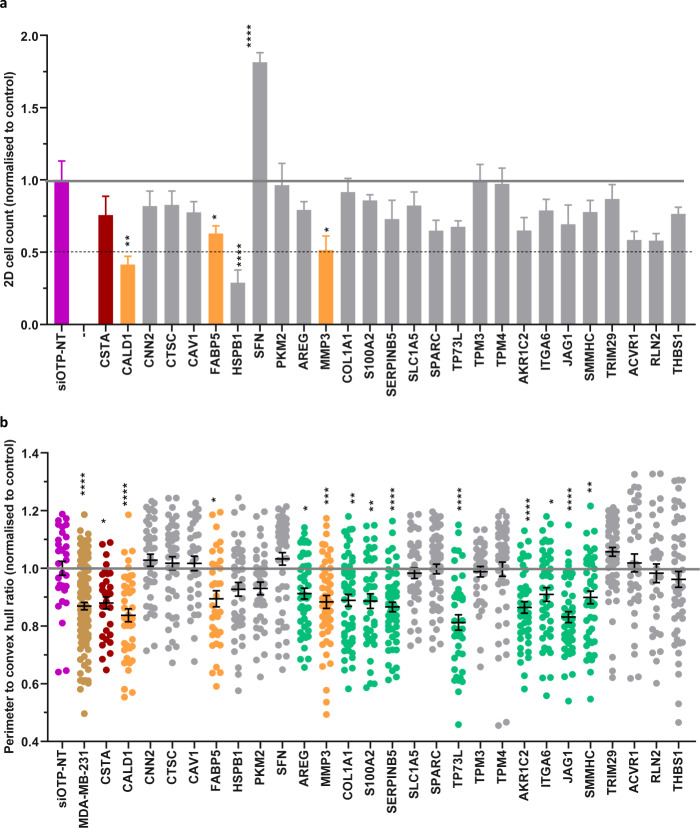
siRNA knockdown of a panel of myoepithelial proteins affects ability to control tumor cells. As for Fig. 4: (**a**) 2D cell count of Hoechst stained N1ME cells 48 hours post-siRNA transfection normalised to siOTP-NT control. Error bars represent SEM across technical replicates. (**b**) Perimeter to convex hull ratio of MDA-MB-231 cells in co-culture with N1ME cells following siRNA transfection. Dots in the graph represent an individual 3D structure/colony growing within the well. siOTP is shown in purple, MDA-MB-231 cells alone are gold, siRNA which has resulted in no change in 2D cell viability but has reduced 2D ‘roundness’ is shown in green and siRNA that has resulted in decreased 2D viability and 3D ‘roundness’ is shown in orange. *p < 0.05, **p < 0.01, ***p < 0.001, ****p < 0.0001. *n* = *2*. Error bars represent SEM.

**Fig. 6:**
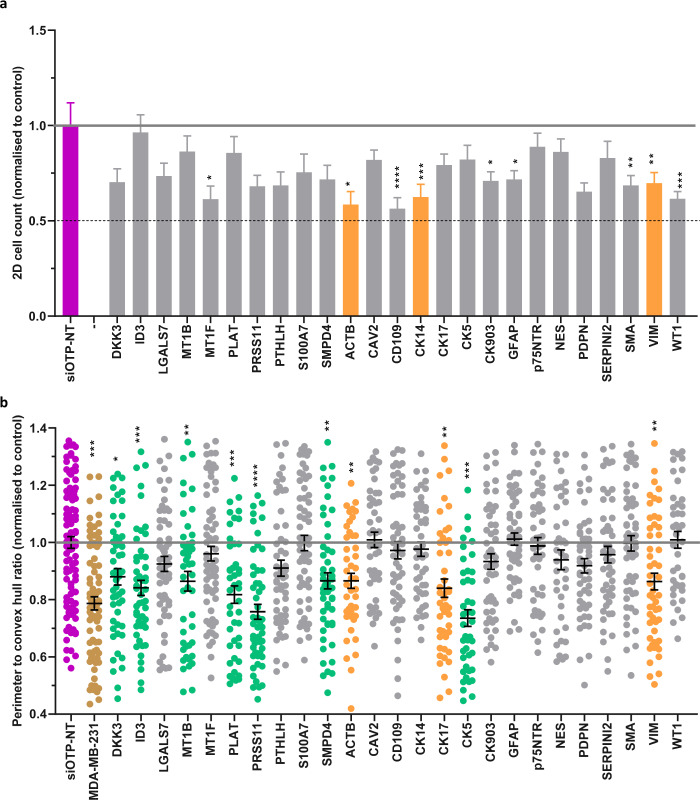
Loss of a second panel of myoepithelial proteins via siRNA knockdown reveals candidate suppressors of tumor invasion. As for Fig. 4: (**a**) 2D cell count of Hoechst stained N1ME cells 48 hours post-siRNA transfection normalised to siOTP-NT control. Error bars represent SEM across technical replicates. (**b**) Perimeter to convex hull ratio of MDA-MB-231 cells in co-culture with N1ME cells following siRNA transfection. Dots in the graph represent an individual 3D structure/colony growing within the well. siOTP is shown in purple, MDA-MB-231 cells alone are gold, siRNA which has resulted in no change in 2D cell viability but has reduced 2D ‘roundness’ is shown in green and siRNA that has resulted in decreased 2D viability and 3D ‘roundness’ is shown in orange. *p < 0.05, **p < 0.01, ***p < 0.001, ****p < 0.0001. *n* = *2*. Error bars represent SEM.

In order to investigate whether other protease inhibitors or adhesion proteins were integral to myoepithelial cell control of tumour cell invasion, we combined high-content siRNA screening with our 3D co-culture system (Fig. 1). We utilized a boutique library of 87 siRNA targeting proteins that have well documented roles in adhesion or protease inhibition and were previously identified in breast myoepithelial cells, or are classical myoepithelial ‘markers’ (Data table, PubChem AID: 1508613). Knockdown of protein expression via siRNA in myoepithelial cells (Fig. 2) was followed by co-culture with tumour cells in our 3D model. This allowed identification of any siRNA-directed gene knockdown “hits” that altered the myoepithelial cell ability to control the invasive phenotype of breast cancer cells (Figs. 3–6). Along with 3D analysis, we have also generated a dataset highlighting the effects that siRNA-mediated knockdown of specific proteins has on myoepithelial cell viability in 2D (Figs. 4–6). The generation of this unique dataset highlighting proteins that play a role in myoepithelial mediated suppression of tumour cell invasion or survival can be utilised by researchers as a basis for further validation and functional exploration of specific genes in the DCIS-IDC transition. The potential of such genes is supported by the observation that one such gene hit was stefin A, a previously validated target at a functional and diagnostic level^5^. Clinical validation and research extending from our findings may reveal novel approaches for individualised patient stratification and treatment in early breast cancer and open the door to new areas of research in the understudied myoepithelial field.

## Methods

### An overview of the work flow is outlined in Figure 1

Briefly, master plates of siRNAs were created and performed on different dates. 2X transfection reagent was transferred across 2 identical plates producing 2 daughter plates with replicates on each half of the plate. One plate was carried through to the 3D co-culture study and the other was used as a reference for siRNA impact on cell viability (in 2D) at the time of embedding in 3D. Replicate wells were measured for cell viability and the cells were embedded manually into 3D culture.

### Cell lines used

The N1ME cell line was derived by Kornelia Polyak as previously described^5^ and cultured in Mammary Epithelial Cell Growth Medium (MEGM; Lonza, CC3151) with Single Quot supplements (Lonza CC-4136). The MDA-MB-231 cell line was maintained in DMEM with 10% FBS. All cell lines were maintained at 37 °C and 5% CO_2_. It should be noted that 3D co-cultures using these cell lines utilised the MEGM media.

### siRNA transfection and 2D imaging

Overview provided in Fig. 1. Master plates of siRNAs were created and screens performed on different dates. For each master plate, target SMARTpool siRNAs, four individual Dharmacon siRNAs targeting each gene pooled per well; siOnTARGET-Plus non-targeting control (OTP-NT); siTOX (control for transfection efficiency – causes cell death) or mock (siRNA buffer only) were freshly plated in the left-hand side of a 96 well plate as a 4X master mix. A Sciclone ALH3000 liquid handling robot (Perkin Elmer) was utilized to transfer 2X master mix to the right-hand side of the 96 well plate, then the entire plate stamped to a 1X copy. This produced 2 daughter plates with replicates on each half of the plate, resulting in 4 wells of siRNA for each gene of interest, OTP-NT, siTOX and mock transfection. The siRNA were reverse transfected into the N1ME cell line using DharmaFECT 3 (Dharmacon, Horizon Discovery) and Opti-MEM (ThermoFisher) at 40 nM final concentration. Briefly, lipid was complexed in Opti-MEM for 5 minutes before addition to siRNA and complexing for a further 20 minutes. 6000 N1ME cells were then added to the siRNA transfection complex in a 96 well plate at 37 °C and 5% CO_2_. Media was changed 24 hours post-transfection. Forty-eight hours post-transfection, cells from one 96 well plate were seeded into 3D culture as described below. As mentioned, cells were transfected in duplicate, and at this time point the duplicate 96 well plate was stained with cell-permeable Hoechst (Thermo Scientific, Cat #33342) to label the nuclei and imaged in high content using the Cellomics ArrayScan VTi or Cell Insight CX7 (Thermo Fisher Scientific) using a 10x objective. Cell nuclei were counted using the inForm software (PerkinElmer) (Figs. 2–6). Aside from the siRNA master plate creation and transfection steps, all other liquid handling steps used a BioTek406 liquid handling workstation (BioTek).

### 3D cell culture

At 48 hours post-transfection and prior to beginning the 3D culture, we took a record of cell viability of the N1ME cells in the replicate plate by imaging using bright field on the Cytation 3 multi-mode plate reader/imager (BioTek) using a 2.5x objective. These images were kept as a reference to relate cell viability to that measured by nuclei staining if needed (data not included). All 3D cultures were performed using a reduced growth factor reconstituted basement membrane matrix, Cultrex® (Trevigen, 3433-005-01) as previously described^5^. In this two-step process, the N1ME siRNA transfected cells and MDA-MB-231 cells were trypsinised in parallel. The entire contents of the siRNA transfected N1ME wells (originally 6000 cells/well seeded pre-transfection and cell viability as indicated in Figs. 4–6) were manually transferred into the individual wells of a 96 well round bottom plate along with 6000 cells per well of MDA-MB-231. For the MDA-MB-231 only control wells (in 3D culture), 6000 cells per well were added to empty wells (no N1ME cells) of the same plate. The round bottom 96 well plate was centrifuged at 350 × g for 5 minutes and cells were resuspended in 40 µl MEGM. Meanwhile, 30 µl neat Cultrex (12–18 mg/ml protein) was laid in a 48 well plate and allowed to solidify at 37 °C for 20 minutes. Following this, 20 µl of the MDA-MB-231 and N1ME cell suspension, or MDA-MB-231 cells alone, were seeded onto Cultrex and allowed to adhere for 60–90 minutes before an overlay of MEGM containing 2% Cultrex was added. This layering technique ensures that the 3D structures are within the same focal plane. This was repeated for the left and right hand sides of the originally transfected siRNA 96 well plate, one half in the morning and one half in the afternoon. Media was changed on day 4 post seeding. On day 7, 3D cultures were stained with a 1/1000 dilution of Hoechst (10 mg/ml stock) added directly into culture media and incubated in the dark for 10 minutes. Cultures were immediately imaged on the ArrayScan VTi (Thermo Fisher Scientific) using a 5x objective, with 5 × 3 fields using bright field, Hoechst.

### 3D cell culture quantification

Brightfield images taken using a 5x objective were stitched together using the Cellomics HCS studio 3 software to give an image of the entire well on day 7 post-seeding. For quantification, images were processed and analyzed using the Fiji distribution of ImageJ^9^ as described by^5^. Briefly, in Fiji, an edge filter was applied followed by an unsharp mask (radius = 4, mask = 0.8) to generate masks of the individual cultures. The image was then blurred using a Gaussian filter (sigma = 4) for accurate thresholding. The threshold was applied and manually adjusted as required, resulting in a binary mask. The mask was filtered by size to remove small debris. Each individual mask surrounding cultures was then measured for its perimeter and convex hull lengths. The convex hull length was then divided by the perimeter length to generate the convex hull to perimeter ratio used for the subsequent data analysis (Figs. 2–6). A value of 1 indicates a smooth object; as the value moves away from 1 towards 0, the number and/or size of the protrusions from the colony is increased. Therefore, the perimeter to convex hull ratio gives an indication of ‘circularity’ i.e. the more circular a colony of cells, the less invasive it is. Whereas when the number or size of protrusions emanating from a colony increases, this decreases the ‘circularity’ and perimeter to convex hull ratio, suggesting a more invasive phenotype than control (siOTP-NT). The perimeter to convex hull ratio was normalized to the circularity seen with the siOTP-NT control from the plate on which the co-culture was seeded. To avoid colonies that may have started as a single cell and therefore may not represent a co-culture, only colonies which had an area greater than 5,000 pixels (33,282 um^2^, calculated using the Image J script) were graphed.

### Western blot analysis

Cell pellets were collected from N1ME cells transfected with OTP-NT or CAV2 siRNA at 48 hours and 7 days post-transfection, N1ME cells transfected with stefin A siRNA 48 hours post transfection, and N1ME cells with permanent stefin A knockdown via TALEN^5^. Cells were lysed, and standard western blot procedure was followed as previously described^5^. Following transfer, membranes were incubated with primary antibodies against stefin A (ab61223, Abcam, Cambridge, UK; 1 µg/ml), GAPDH (8884, D16H11, Cell Signalling, MA, USA; 1:10,000 dilution), CAV2 (ab79397, Abcam; 1:3,000 dilution) or anti-β-actin (A22280, Sigma-Aldrich, MO, USA; 1:10,000 dilution).

### Statistical analysis

Statistical analyses were conducted using the data analysis software package within GraphPad Prism v7 for Windows (GraphPad Software). One-way ANOVA followed by Dunnett’s multiple comparison test was used to calculate statistical differences in distribution of gene targeted myoepithelial siRNA co-cultures compared to the siOTP-NT controls in co-culture or to compare biological replicates across different runs. All statistical tests were on raw data prior to normalization to control. 3D co-cultures were screened in duplicate (therefore two biological replicates), however the data presented represents one biological replicate, all data images are uploaded and available. Error bars indicate SEM unless otherwise stated and each data point represents individual 3D spheroid structures.

## Data Records

### Data record 1

2D images of Hoechst stained wells 48 hours post transfection imaged on the CX7. Images are provided as fields for each well that can be analysed individually or stitched together to give an overview of the whole well. These images were used to determine 2D cell viability. Images are labelled with plate (run) number, MDA-MB-231, siRNA target gene and numbered 1 through to 8/16 depending on how many fields available (8 for Arrayscan7, 16 for CX7). 2 wells for each siRNA target were imaged i.e. fields 1–4 (or 1–8) from one well, fields 5–8 (or 9–16) from another well. Uploaded to BioStudies, accession number: S-BSST514^10^.

### Data record 2

Whole well montaged images of 3D cultures taken in brightfield using the ArrayScan VTi. Fields have been stitched together to give an overview of the whole well. These images were run through Image J to determine the perimeter to convex hull ratio. Images are labelled as plate (run) number, siRNA target gene. Uploaded to BioStudies, accession number: S-BSST514^10^.

### Data record 3

The entire screen dataset with 2D and 3D quantification. All raw data (perimeter to convex hull ratio) and normalised data (normalised to siOTP-NT on same run) from each plate is located in individual tabs in the table. Uploaded to BioStudies, accession number: S-BSST514^10^.

### Data record 4

Average normalised 3D quantification (perimeter to convex hull ratio) and 2D cell counts along with 3D hits, reagent ID, substance ID, short and long gene names and run number for each siRNA target has been uploaded to PubChem. PubChem AID: 1508613^11^.

## Technical Validation

Multiple controls were included on each plate to ensure quality data collection and reproducibility across screen weeks: a non-targeting control (siOTP-NT, 1 well/3D plate and 2 wells/2D plate), a lethal control (siTOX, 2 wells/2D plate - greater than 99% of cells were killed following transfection), a mock control (siRNA buffer alone 1 well/3D plate and 2 wells/2D plate), and single cell-specific control (MDA-MB-231 cells alone 4 wells/3D plate). Data was normalised to the average of the siOTP-NT well(s) on a per plate basis i.e. all samples in run 1 were normalised to siOTP-NT in run 1. Effect of siRNA control on 2D cell viability and spread of controls in 3D culture can be seen in Fig. 2. Repeat validation of 3 hits, HSPB1, Tp73L (p63) and ACTB were done on different runs (Fig. 2). There was no statistical difference seen in the repeat hit suggesting that the data from this model is reproducible. Permanent knockdown of stefin A (CSTA) using TALENs was previously completed^5^ and this data confirms the knock-down phenotype illustrated here with siRNA transfection, as illustrated in Fig. 2h,i. Western blot of siRNA knockdown at 48 hours and 7 days post siRNA transfection is shown for CAV2 (Fig. 2j).

## Usage Notes

For ease of usability, we have classified the targets into functional groups: adhesion proteins, protease inhibitors or myoepithelial proteins. Data record 3 and 4 identifies these groups and associated references.

In these 3D co-cultures, an increase in invasiveness over control myoepithelial cells (siOTP-NT) suggests that the gene targeted by the siRNA has a role in suppression of breast cancer cell invasion. On the contrary, a decrease in invasiveness suggests that the gene targeted normally promotes invasion. Anything that was statistically different from the siOTP-NT from the relevant run is indicated as a hit in data record 3 and 4.

For every siRNA target, 2 wells of N1ME cells were transfected and then set up in two separate 3D plates i.e. Run 1 A and 1B. All data shown in the figures is from one plate i.e. only run A or B however all raw data from both plates is provided in the files online.

Knockdown of ACTB was consistent with published outcomes, where loss is known to have an effect on cell viability and motility^12^. When knocked down in the N1ME cells there was a decrease in 2D viability and 3D control of tumor cells. The TIMPs (1, 2, 3 & 4) are negative regulators of pubertal ductal elongation^13,14^, whereby knockdown of TIMPs has been shown to increase ductal expansion and number of ducts. This role of TIMPs matches well with our results, where we see an increase in the capacity of myoepithelial cells to control invasive tumor cells on day 7 when they lack expression of TIMP2, TIMP3 and TIMP4. For the majority of targets screened, this dataset represents the first time that a myoepithelial specific role has been reported in the literature.

## Data Availability

For access to the code used to calculate the perimeter to convex hull ratio: 10.26180/5efec4822e873^15^.
